# The Book of Prescriptions

**Published:** 1855-12

**Authors:** 


Art. VII.—*The Book of Prescriptions: Containing* 2900 *Prescriptions, collected from the practice of the most eminent physicians and surgeons, English and Foreign. Comprising also a compendious history of the Materia Medica of all countries, alphabetically arranged; and lists of the doses of all official or established preparations.* By Henry Beasley. Philadelphia: Lindsay & Blakiston. 8vo. pp. 369. 1855.

The editor of the above handsome volume has compiled a most comprehensive and yet an entirely portable work, in which both physician and druggist may find, under its appropriate head, almost every remedy in use, and the manner in which it may be best administered, or combined with other remedies, in the treatment of various diseases. A short description of each medicine is given and the whole arranged alphabetically. To both the prescriber and compounder of drugs the Book of Prescriptions can but prove useful. We have already found it a material assistance in many respects relating to the combination of doses of those medicines which have been more recently brought into notice.

